# The effect of placental transfusion on hemodynamics in premature newborns: a randomized controlled trial

**DOI:** 10.1007/s00431-022-04619-0

**Published:** 2022-09-21

**Authors:** Marwa Mohamed Farag, Mohamed Alaa Eldin Hassan Thabet, Ali Mohamed Abd-Almohsen, Heba Idris Abudeif Mohammed Ibrahim

**Affiliations:** Pediatric Department, Alexandria University, Alexandria, Egypt

**Keywords:** Fractional shortening, Superior vena cava flow, Delayed cord clamping, Cord milking

## Abstract

Despite of growing evidence of the beneficial effects of placental transfusion techniques, there is no available sufficient data about their effects on vulnerable hemodynamics and myocardium of premature infants. The purpose of this work is to study ventricular functions and hemodynamics after applying different placental transfusion techniques, delayed cord clamping (DCC), cut cord milking (C-UCM), and intact cord milking (I-UCM). Sixty-four infants delivered whether by C-section or vaginal delivery were randomly assigned to undergo C-UCM (20–30 cm), I-UCM (3–4 strippings), and DCC (30–60 s). Functional echocardiography was done on day 1 and day 3 of life for 57 infants. Primary outcome variable was superior vena cava flow measurement in infants having placental transfusion in the first 24 h of life and between 64 and 72 h. Secondary outcomes were other echocardiographic and clinical hemodynamic parameters, and biventricular functions in those infants. Of a total 196 preterm infants ≤ 32 weeks delivered in the study period, from January 2021 to August 2021, 57 infants were eligible and survived till the second examination. They were randomly assigned to the three groups. Neonates randomly assigned to DCC had significantly higher superior vena cava flow and lower right ventricular systolic function in the first 24 h of life. This finding vanished at day 3. Neonates undergone different methods of placental transfusions had similar hemoglobin, admission temperature, and mean blood pressure in the first 24 h of life.

*Conclusion*: Despite their potential benefits, placental transfusions have shown to alter the hemodynamics and adversely affect myocardial function of premature neonates.

*Trial registration*: This trial was registered in the clinical trial gov NCT04811872.

**Table:** 

**What is Known:** *• Placental transfusion techniques might have benefits regarding prematurity- related morbidities and mortality.*	
**What is New:** *• Placental transfusion might adversely affect the myocardium and alter hemodynamics in premature infants.*

**Supplementary Information:**

The online version contains supplementary material available at 10.1007/s00431-022-04619-0.

## Introduction

Transition from fetal to neonatal life is the most complex life time event of a human being. Prematurity is one of the factors that hamper smooth transition and placental transfusion is one of the techniques being utilized to facilitate adequate transition [1]. In addition to providing adequate blood volume, and improving pulmonary flow, pulmonary vascular resistance, left ventricular preload, and tissue oxygenation, placental transfusion techniques provide various benefits that extend beyond the transition period such as decreasing the incidence of necrotizing enterocolitis (NEC), intraventricular hemorrhage (IVH), late onset sepsis, respiratory support days, inotropes and transfusion needs, and improving hemoglobin levels, iron stores, and neurodevelopmental outcome. Simply, placental transfusion might help in solving the challenging problems of prematurity, thereby improving the morbidities and mortality of preterm infants [2–11]. There are 3 types of placenta transfusions that are variable in timing, duration, and amount of blood transfused including cut-umbilical cord milking (C-UCM), intact umbilical cord milking (I-UCM), and delayed cord clamping (DCC) [12].

There is a paucity of studies that compare those different techniques regarding their effect on cardiovascular parameters. In the current work, we aim at studying the effect of those different techniques on hemodynamics and cardiac function of premature infants.

### Aim of the work

Studying the effects of different placental transfusion techniques on the hemodynamics and cardiac functions of premature neonates ≤ 32 weeks.

## Method

This three-armed parallel randomized control trial was conducted at the Alexandria University Maternity Hospital (AUMH), from January 2021 to August 2021. Approval from Institute Ethics Committee and parental consents were obtained. This trial was registered in the clinical trial gov NCT04811872.

### Randomization and blinding

Randomization into the 3 groups was done using random permuted blocks of 6 and 9 size prepared by a person not involved in the study. Allocation into the three groups was done using serially numbered opaque and sealed envelopes by neonatology residents attending deliveries. The obstetricians were informed about plan of cord management before delivery. Blinding was not applicable at the stage of placental transfusion due to nature of the intervention, while at the stage of scanning, the operator was blinded to the state of placental transfusion.

### Sample size calculation

A minimal total sample size of 57 patients (19 per group) is needed to detect a difference in mean superior vena cava flow (SVCF) among three groups of preterm infants with different methods of placental transfusion (intact umbilical cord milking, cut-umbilical cord milking, and delayed cord clamping) to study the effects of these methods on hemodynamics and neonatal outcomes in premature neonates with assumed group means of 93, 70, and 81 ml/kg/min, respectively, using group standard deviations of 24, 22, and 29 ml/kg/min, one-way ANOVA test, a significance level of 0.05, and 80% power (PASS program version 20) [13].

### Study population

**Fig. 1:**
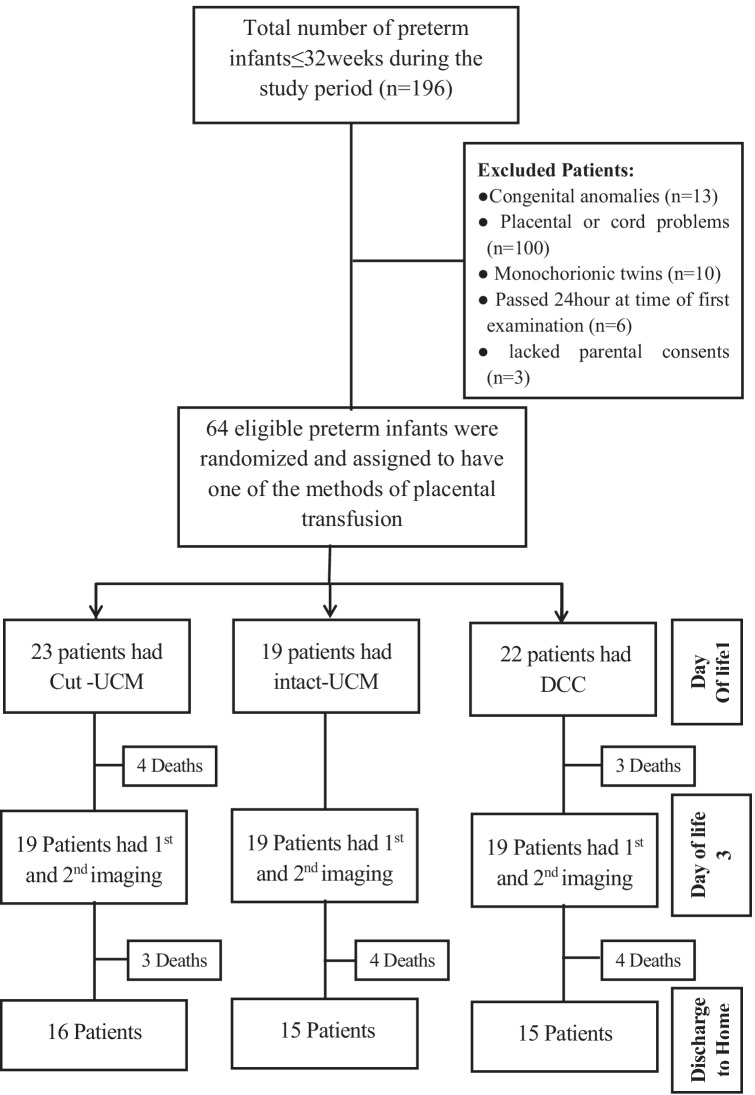
Consort flow diagram of participants in the study

At the study period, 196 preterm infants ≤ 32 weeks were born in AUMH. This is a tertiary center that receives critically ill pregnant women from three governments. Only 64 were eligible and randomized to three groups. Patients with congenital anomalies (*n* = 13), placental or cord problems (*n* = 100), passed 24 h at time of first examination (*n* = 6), or lacked parental consents (*n* = 3) were excluded from the study. Of 64 infants who had one of placental transfusion methods and first imaging, fifty-seven infants lived till time of the second examination (Fig. 1).

### Interventions

The study was designed to be carried out in 2 steps. As a first step, 64 pregnant women who are expected to deliver at or before 32 0/7 weeks were randomly assigned to 3 experimental groups: cut-umbilical cord milking (C-UCM), intact umbilical cord milking (I-UCM), and delayed cord clamping (DCC).

A cut-umbilical cord milking entails clamping and cutting a long segment (20–30 cm) of the umbilical cord immediately after birth and handing it over to the attending neonatologist, who will untwist the cord and milk the entire contents into the baby three times.

Intact umbilical cord milking (I-UCM): Umbilical cord milking was performed by holding the newborn between the maternal thighs. The cord was pinched between 2 fingers as close to the placenta as possible and milked to the infants’ side over a 2-s duration, then it was released and allowed to refill with blood for a brief 1- to 2-s pause between each milking motion. This was repeated for 3 times. Group of delayed cord clamping (DCC) in which infants were placed between the maternal thigh below the level of placenta and waiting 30–60 s before clamping the cord. The resident physician attending the delivery recorded the time elapsed from when the infant was delivered until the time the umbilical cord was clamped by the obstetrician to make sure that time exceeded 30 s [12].

Second step, a prospective cohort study, in which the operator was blinded to state of placental transfusions. A model of GE Vivid iq premium was used for functional echocardiography (FE). There was a GE 12S-RS probe with a frequency range of 5–11 MHz. An examination was performed using 2D, M-mode, color Doppler, continuous wave, and pulsed wave (PW) Dopplers. Doppler volumetric measurements of the superior vena cava flow, left ventricular output (LVO), and right ventricular output (RVO) were measured using Evans and Kluckow methodology [14, 15]. The velocity time integral (VTI) was calculated from the Doppler velocity tracings and averaged over 5 consecutive cardiac cycles in case of SVCF and 3 consecutive cycles in case of RVO and LVO estimations. The heart rate was measured from the peak-to-peak intervals of the Doppler velocity time signals. The duct was measured at its narrowest point, before its entry into the main pulmonary artery. Ductus arteriosus (DA) size, when indexed to the patient’s weight, > 1.4 mm/kg, together with left atrium to aortic root ratio (LA/AO) ratio were considered as markers of hemodynamically significant DA.

Left ventricular systolic and diastolic functions were assessed using fraction shortening (FS) and ejection fraction (EF), and mitral E/A ratio, respectively. Systolic right ventricular function was assessed using tricuspid annular plane systolic excursion (TAPSE) [16].

Cranial sonography was done with a GE 8C-RS probe with a frequency range of 3.5–10 MHz of A model GE Vivid iq premium. Patients were scanned regarding intraventricular hemorrhage (IVH) status and ACA (anterior cerebral artery) velocities; PSV (peak systolic velocity) and EDV (end diastolic velocity), and RI (resistive index) in the first 24 h [17]. On day 3, another cranial scan was performed to screen for IVH without measuring cerebral Doppler velocities.

Primary outcome variable was SVCF in the first day and at day 3 after the procedure of placental transfusion. Secondary outcome variables were other hemodynamic measures such as LVO, RVO, DA, LA/Ao ratio and ACA velocities, cardiac function parameters such as FS, TAPSE and E/A ratio, as well as clinical parameters such as IVH, admission temperature, initial hemoglobin, mean blood pressure, and heart rate on the first day of life. A second FE was conducted on the third day of life for all living patients.

Data were fed to the computer and analyzed using IBM SPSS software package version 20.0. The Kolmogorov–Smirnov test was used to verify the normality of distribution. Qualitative data were described using number and percent. Quantitative data were described using range (minimum and maximum), mean, and standard deviation, median and interquartile range (IQR). Significance of the obtained results was judged at the 5% level. Chi-square test, Monte Carlo correction, *F*-test (ANOVA), and Kruskal Wallis test were used for comparison between the three groups regarding different variables.

## Results

Among the 64 infants randomly assigned to the three groups, 19 were assigned to the I-UCM group, 23 to the C-UCM group, and 22 to the DCC group. Seven patients did not survive to the second examination on third day of life (Fig. 1). In all 57 patients, a functional echocardiography was conducted between 6 and 22 h, and a second one was performed between 64 and 72 h of life. In DCC group, cord clamping was delayed for a period of 32–50 s, with a median of 43 s and a mean of 41.32 ± 5.7 s.

In Table 1, no significant differences were found between the studied groups in terms of demographic characteristics, maternal risk factors, or resuscitation data. Vital signs, laboratory parameters, ventilation needs, and inotropic support did not differ significantly between studied groups (Table 2).

**Table 1:** Demographic information, maternal risk factors, and resuscitation data for each studied group

		Placental transfusion techniques			Test of sig	*p*
		Cut CM	Intact CM	DCC		
Sex						
Male		9 (47.4%)	12 (63.2%)	10 (52.6%)	χ^2^ = 0.990	0.610
Female		10 (52.6%)	7 (36.8%)	9 (47.4%)		
GA						
Min.–max		27.0–32.0	27.0–32.0	27.0–32.0	*H* = 4.174	0.124
Mean ± SD		30.37 ± 1.42	29.79 ± 1.44	29.47 ± 1.43		
Median (IQR)		30.0 (30.0–32.0)	30.0 (29.0–31.0)	29.0 (29.0–30.0)		
BWT						
Min.–Max		0.75–1.45	0.57–1.50	0.60–1.40	*F* = 2.046	0.139
Mean ± SD		1.14 ± 0.17	1.04 ± 0.25	0.99 ± 0.24		
Maternal age					*F* = 0.360	0.699
Min.–Max		18.0–43.0	18.0–39.0	15.0–42.0		
Mean ± SD		26.84 ± 6.99	27.37 ± 5.91	25.58 ± 7.09		
Fertilization	Spontaneous	15 (78.9%)	14 (73.7%)	15 (78.9%)	χ^2^ = 3.845	^*MC*^*p* = 0.474
	Ovulation induction	0 (0.0%)	3 (15.8%)	2 (10.5%)		
	ICSI	4 (21.1%)	2 (10.5%)	2 (10.5%)		
MOD	NVD	5 (26.3%)	8 (42.1%)	9 (47.4%)	χ^2^ = 1.925	0.382
	CS	14 (73.7%)	11 (57.9%)	10 (52.6%)		
Multiplicity	Single	11 (57.9%)	7 (36.8%)	11 (57.9%)	χ^2^ = 2.246	0.325
	Multiple	8 (42.1%)	12 (63.2%)	8 (42.1%)		
Antenatal care	No	0 (0.0%)	2 (10.5%)	0 (0.0%)	χ^2^ = 4.145	^*MC*^*p* = 0.327
	Yes	19 (100.0%)	17 (89.5%)	19 (100.0%)		
Antenatal steroids	No	4 (21.1%)	7 (36.8%)	9 (47.4%)	χ^2^ = 2.297	0.231
	Yes	15 (78.9%)	12 (63.2%)	10 (52.6%)		
Anesthesia	No	5 (26.3%)	8 (42.1%)	9 (47.4%)	χ^2^ = 4.123	^*MC*^*p* = 0.428
	Spinal	14 (73.7%)	10 (52.6%)	10 (52.6%)		
	General	0 (0.0%)	1 (5.3%)	0 (0.0%)		
Anemia	No	9 (47.4%)	9 (47.4%)	10 (52.6%)	0.140	0.932
	Yes	10 (52.6%)	10 (52.6%)	9 (47.4%)		
PET	No	14 (73.7%)	12 (63.2%)	14 (73.7%)	0.671	0.715
	Yes	5 (26.3%)	7 (36.8%)	5 (26.3%)		
DM	No	17 (89.5%)	15 (78.9%)	19 (100.0%)	4.471	^*MC*^*p* = 0.153
	Yes	2 (10.5%)	4 (21.1%)	0 (0.0%)		
PROM	No	8 (42.1%)	9 (47.4%)	5 (26.3%)	1.925	0.382
	Yes	11 (57.9%)	10 (52.6%)	14 (73.7%)		
PTLP	No	4 (21.1%)	3 (15.8%)	3 (15.8%)	0.243	^*MC*^*p* = 0.999
	Yes	15 (78.9%)	16 (84.2%)	16 (84.2%)		
Maternal infection	No	9 (47.4%)	10 (52.6%)	15 (78.9%)	4.519	0.104
	Yes	10 (52.6%)	9 (47.4%)	4 (21.1%)		
Diastolic flow	Normal	14 (73.7%)	17 (89.5%)	14 (73.7%)	1.900	^*MC*^*p* = 0.549
	Abnormal	5 (26.3%)	2 (10.5%)	5 (26.3%)		
CTG	Normal	16 (84.2%)	16 (84.2%)	18 (94.7%)	1.303	^*MC*^*p* = 0.671
	Abnormal	3 (15.8%)	3 (15.8%)	1 (5.3%)		
Resuscitation	Initial steps	15 (78.9%)	15 (78.9%)	14 (73.7%)	χ^2^ = 0.545	^*MC*^*p* = 1.000
	PPV (mask)	3 (15.8%)	3 (15.8%)	3 (15.8%)		
	PPV (ETT)	1 (5.3%)	1 (5.3%)	2 (10.5%)		
APGAR at 1 min						
Min.–max		2.0–8.0	5.0–7.0	5.0–7.0	*H* = 0.100	0.951
Mean ± SD		6.0 ± 1.37	6.16 ± 0.83	6.26 ± 0.65		
APGAR at 5 min						
Min.–max		6.0–9.0	7.0–9.0	7.0–9.0	*H* = 1.233	0.540
Mean ± SD		8.16 ± 1.07	8.21 ± 0.79	8.05 ± 0.52		

*χ*^*2*^ chi-square test, *F* one-way ANOVA, *H* Kruskal Wallis test, *MC* Monte Carlo, *p p* value for comparing between the three studied groups, *GA* gestational age, *BWT* birth weight, *MOD* mode of delivery, *CS* cesarean section, *NVD* normal vaginal delivery, *PET* preeclampsia, *DM* diabetes mellitus, *PROM* prolonged rupture of membranes, *PTLP* preterm labor pain, *CTG* cardio-tocography

Statistically significant at *p* ≤ 0.05

**Table 2:** The initial vital signs, laboratory parameters, ventilation needs, and inotropic support in each group in the 1st 24 h

		Placental transfusion techniques			Test of sig	*p*
		Cut CM	Intact CM	DCC		
Temperature						
Min.–max		30.50–38.0	34.50–38.0	34.20–37.40	*H* = 1.406	0.495
Mean ± SD		35.73 ± 1.66	36.32 ± 0.96	36.04 ± 0.85		
HR beat/min						
Min.–max		140.0–170.0	130.0–170.0	130.0–180.0	*H* = 2.187	0.335
Mean ± SD		153.16 ± 10.03	156.05 ± 9.51	152.74 ± 11.84		
RR breath/min						
Min.–max		40.0–75.0	40.0–70.0	40.0–65.0	*H* = 0.465	0.793
Mean ± SD		56.84 ± 10.03	54.47 ± 10.39	55.79 ± 6.72		
CRT sec						
Min.–max		2.0–4.0	2.0–3.0	2.0–3.0	*H* = 0.100	0.951
Mean ± SD		2.26 ± 0.56	2.26 ± 0.45	2.26 ± 0.45		
MABP mmHg						
Min.–max		14.0–71.0	16.0–43.0	16.0–44.0	*H* = 3.225	0.199
Mean ± SD		29.68 ± 12.90	27.32 ± 6.81	30.95 ± 6.94		
SPO2						
Min.–max		93.0–100.0	92.0–100.0	92.0–100.0	*H* = 0.063	0.969
Mean ± SD		98.79 ± 2.04	98.79 ± 2.07	98.79 ± 2.23		
HB g/dl						
Min.–max		13.0–19.20	13.0–19.60	10.0–18.80	*F* = 0.727	0.488
Mean ± SD		16.06 ± 1.57	16.04 ± 1.78	15.40 ± 2.31		
Median (IQR)		16.40 (14.40–17.0)	16.0 (14.50–17.30)	16.10 (13.50–17.40)		
WBC × 10^3^ cell/dl						
Min.–max		3.10–22.20	3.10–45.60	3.50–80.0	*H* = 0.202	0.904
Mean ± SD		10.78 ± 4.43	12.24 ± 9.32	16.05 ± 17.84		
Median (IQR)		10.30 (7.90–13.10)	10.40 (7.30–13.50)	11.0 (5.30–18.30)		
CRP						
Min.–max		0.0–1.0	0.0–1.0	0.0–1.0	*H* = 2.220	0.330
Mean ± SD		0.16 ± 0.37	0.32 ± 0.48	0.37 ± 0.50		
Median (IQR)		0.0 (0.0–0.0)	0.0 (0.0–1.0)	0.0 (0.0–1.0)		
BC	Negative	18 (94.7%)	18 (94.7%)	18 (94.7%)	χ^2^ = 0.00	^*MC*^*p* = 1.000
	Positive	1 (5.3%)	1 (5.3%)	1 (5.3%)		
Ventilation mode	Non-invasive	18 (94.7%)	17 (89.5%)	17 (89.5%)	χ^2^ = 0.438	^*MC*^*p* = 1.000
	Invasive	1 (5.3%)	2 (10.5%)	2 (10.5%)		
Inotropes	No	17 (89.5%)	16 (84.2%)	18 (94.7%)	χ^2^ = 1.118	
	Yes	2 (10.5%)	3 (15.8%)	1 (5.3%)		

*χ*^*2*^ chi-square test, *F* one-way ANOVA, *H* Kruskal Wallis test, *MC* Monte Carlo, *p p* value for comparing between the three studied groups, *HR* heart rate, *RR* respiratory rate, *CRT* capillary refill time, *MABP* mean arterial blood pressure, *SPO2* oxygen saturation, *BC* blood culture

Statistically significant at *p* ≤ 0.05

No significant differences were found among the three groups regarding PDA size, direction, and being hemodynamically significant. SVCF, the primary outcome, was significantly higher in the DCC group than I-UCM and C-UCM groups with mean values 120.29 ± 43.11, 83.5 ± 34.83, and 97.44 ± 30.27, respectively, *p* value 0.013 (Table 3). While after day 3, no significant differences were found among the three studied groups (Table 4).

**Table 3:** Echocardiographic parameters in the 3 groups; myocardial function, SVCF, LA/Ao ratio, and PDA parameters in the first 24 h

ECHO parameters	Cut CM (*n* = 19)		Intact CM (*n* = 19)		DCC (*n* = 19)		Test of sig (*p*)
**SVC flow (ml/kg/min)**							*H* = 8.629 *p* = 0.013*
Mean ± SD	83.5 ± 34.83		97.44 ± 30.27		120.29 ± 43.11		
Median (min–max)	72.4 (36.3–173.7)		91.7 (60–170)		99.0 (55–183.6)		
Significance bet. groups	*p*1 = 0.386, *p*2 = 0.01*, *p*3 = 0.469						
**LA/AO ratio**							*H* = 0.210 *p* = 0.9
Mean ± SD	1.28 ± 0.32		1.18 ± 0.15		1.22 ± 0.24		
Median (min–max)	1.2 (0.8–2.0)		1.2 (1.0–1.5)		1.14 (0.97–1.7)		
**EF (%)**							*H* = 0.896 *p* = 0.639
Mean ± SD	48.84 ± 7.17		50.0 ± 7.51		47.32 ± 7.82		
Median (min–max)	50.0 (37.0–60.0)		51.0 (34.0–64.0)		46.0 (29.0–61.0)		
**FS (%)**							*H* = 1.026 *p* = 0.599
Mean ± SD	22.37 ± 3.92		22.95 ± 4.17		21.53 ± 4.31		
Median (min–max)	23.0 (16.0–29.0)		23.0 (14.0–31.0)		21.0 (12.0–30.0)		
**LVO (ml/kg/min)**							*H* = 4.194 *p* = 0.123
Mean ± SD	192.24 ± 58.18		162.16 ± 41.0		216.15 ± 89.8		
Median (min–max)	177.7 (110.5–311)		159 (78.8–259.4)		203.8 (83.8–397)		
**RVO (ml/kg/min)**							*H* = 4.726 *p* = 0.094
Mean ± SD	270.32 ± 69.52		327.36 ± 104.55		331.64 ± 89.4		
Median (min–max)	263 (155.8–429)		336.6 (161–538.8)		335.8 (191.4–463.3)		
**E/A ratio**							*H* = 3.910 *p* = 0.142
Mean ± SD	1.04 ± 0.21		0.96 ± 0.12		0.92 ± 0.16		
Median (min–max)	1.01 (0.8–1.74)		0.92 (0.7–1.27)		0.9 (0.57–1.17)		
**TAPSE: cm**							*H* = 9.918 *p* = 0.007*
Mean ± SD	0.53 ± 0.07		0.47 ± 0.08		0.45 ± 0.06		
Median (min–max)	0.5 (0.4–0.6)		0.5 (0.4–0.6)		0.4 (0.4–0.6)		
Significance bet. groups	*p*1 = 0.072, *p*2 = 0.007*, *p*3 = 1.0						
	No	%	No	%	No	%	
PDA closure							^*MC*^*p* = 0.298
Closed	4	21.1	10	52.6	8	42.1	
Non hs-PDA	9	47.3	6	31.6	5	26.3	
hs-PDA	6	31.6	3	15.8	6	31.6	
Size of PDA (mm)							*H* = 5.076 *p* = 0.079
Mean ± SD	1.31 ± 0.95		0.67 ± 1.01		1.21 ± 1.1		
Shunt direction							^*MC*^*p* = 0.113
None	4	21.1	10	52.6	7	36.8	
Bidirectional	0	0.0	1	5.3	0	0.0	
Left to right restrictive	8	42.1	6	31.6	4	21.1	
Left to right non-restrictive	7	36.8	2	10.5	8	42.1	

*H* Kruskal Wallis test, pairwise comparison was done using Bonferroni test, *p p* value between the three groups, *p1 p* value between cut-UCM and intact-UCM, *p2 p* value between cut-UCM and DCC, *p3 p* value between intact-UCM and DCC, *SVCF* superior vena cava flow, *LVO* left ventricular output, *RVO* right ventricular output, *PDA* patent ductus arteriosus, *LA/Ao* left atrium to aortic root ratio, *TAPSE* tricuspid annular plane systolic excursion, *EF* ejection fraction; *FS*, fraction shortening

*Significant (*p* ≤ 0.05)

**Table 4:** Echocardiographic parameters in the 3 groups; myocardial function, SVCF, LA/Ao ratio, and PDA parameters after 72 h

ECHO parameters	Cut CM (*n* = 19)		Intact CM (*n* = 19)		DCC (*n* = 19)		Test of sig. (*p*)
**SVC flow (ml/kg/min)**							*H* = 0.459 *p* = 0.79
Mean ± SD	92.92 ± 24.99		96.4 ± 40.9		103.995 ± 40.9		
Median (min–max)	98.7 (44.4–138)		82.5 (29–180.7)		96.5 (46.3–220)		
**LA/AO ratio**							*H* = 2.2 *p* = 0.33
Mean ± SD	1.14 ± 0.16		1.1 ± 0.21		1.23 ± 0.3		
Median (min–max)	1.1 (1–1.47)		1.07 (0.8–1.8)		1.1 (0.86–2.2)		
**EF (%)**							*H* = 0.014 *p* = 0.99
Mean ± SD	50.05 ± 10.2		50.05 ± 8.99		51 ± 13.7		
Median (min–max)	51 (30–70.0)		49 (34.0–68)		50 (30–77)		
**FS (%)**							*H* = 0.13 *p* = 0.937
Mean ± SD	23.15 ± 5.7		22.7 ± 5.18		23.8 ± 8.4		
Median (min–max)	23.0 (13–36)		22 (14.0–34)		23 (12.0–42)		
**LVO (ml/kg/min)**							*H* = 1.47 *p* = 0.48
Mean ± SD	181.9 ± 48.3		169.8 ± 72		194.59 ± 87.9		
Median (min–max)	171 (112.6–313.3)		160 (71.7–360)		183.3 (90–478.5)		
**RVO (ml/kg/min)**							*H* = 4.03 *p* = 0.133
Mean ± SD	309.2 ± 79.4		401.7 ± 162.47		345.3 ± 97.84		
Median (min–max)	306.6 (191.4–468)		397.6 (124.4–759)		354 (196–583.7)		
**E/A ratio**							*H* = 1.8 *p* = 0.39
Mean ± SD	1 ± 0.11		0.98 ± 0.14		0.96 ± 0.25		
Median (min–max)	1 (0.8–1.26)		1 (0.6–1.26)		0.9 (0.6–1.7)		
**TAPSE: cm**							*H* = 5.7 *p* = 0.058
Mean ± SD	0.5 ± 0.06		0.44 ± 0.08		0.48 ± 0.06		
Median (min–max)	0.5 (0.4–0.6)		0.5 (0.2–0.5)		0.5 (0.4–0.6)		
	No	%	No	%	No	%	
**PDA closure**							^*MC*^*p* = 0.337
Closed	15	78.9	19	100	15	78.9	
Non hs-PDA	3	15.8	0	0	1	5.3	
Hs-PDA	1	5.3	0	0	3	15.8	
Size OF PDA (mm)							*H* = 1.7 *p* = 0.19
Mean ± SD	1.2 ± 0.46				1.8 ± 0.78		
Median (min–max)	1.25 (0.6–1.7)				1.8 (1–2.9)		
Shunt direction							^*MC*^*p* = 0.54
None	15	78.9	19	100	15	78.9	
Bidirectional	0	0.0	0	0	2	10.5	
Left to right restrictive	4	21.1	0	0	0	0.0	
Left to right non-restrictive	0	0.0	0	0	2	10.5	

*H* Kruskal Wallis test, ^*MC*^*p* Monte Carlo exact probability, *SVCF* superior vena cava flow, *LVO* left ventricular output, *RVO* right ventricular output, *PDA* patent ductus arteriosus, *LA/Ao* left atrium to aortic root ratio, *TAPSE* tricuspid annular plane systolic excursion, *EF* ejection fraction, *FS* fraction shortening

TAPSE was significantly lower in DCC than in the other two groups. The second imaging, however, did not reveal any significant differences. Other echocardiographic parameters did not show significant differences between the groups in either scan. Table 5 shows no significant differences among studied groups regarding IVH occurrence, ACA-RI, and cerebral blood flow velocities (PSV and EDV) in the first 24 h. On the second cranial scan, only two patients had progressed from IVH grade I to IVH grade III in the cut-UCM group, although there were still no significant differences among the three groups.

**Table 5:** Intraventricular hemorrhage status, ACA velocities, and resistive index in the first 24 h

Cranial ultrasound parameters	Cut CM (*n* = 19)		Intact CM (*n* = 19)		DCC (*n* = 19)		Test of significance (*p*)
	No	%	No	%	No	%	
IVH							^*MC*^*p* = 1.0
Yes	3	15.8	2	10.5	3	15.8	
No	16	84.2	17	89.5	16	84.2	
Right IVH							
No IVH	16	84.2	17	89.5	17	89.5	^*MC*^*p* = 1.0
IVH grade 1	2	10.5	2	10.5	2	10.5	
IVH grade 2	1	5.3	0	0.0	0	0.0	
Left IVH							^*MC*^*p* = 1.0
No IVH	18	94.7	19	100	18	94.7	
IVH grade 1	1	5.3	0	0.0	1	5.3	
RI							*H* = 1.058 *p* = 0.589
Mean ± SD	0.74 ± 0.09		0.72 ± 0.08		0.73 ± 0.10		
Median (min–max)	0.73 (0.54–0.90)		0.7 (0.58–0.85)		0.71 (0.52–0.94)		
ACA velocity PS (cm/s)							*H* = 1.483 *p* = 0.476
Mean ± SD	18.62 ± 4.16		16.8 ± 3.89		19.14 ± 7.81		
Median (min–max)	19.5 (10.6–24.51)		17.68 (10.8–23.5)		18.7 (9.24–33.7)		
ACA velocity ED (cm/s)							*H* = 0.452 *p* = 0.798
Mean ± SD	5.05 ± 2.46		4.73 ± 1.78		4.75 ± 2.41		
Median (min–max)	4.54 (2.53–11.38)		4.38 (2.24–8.36)		4.57 (2.14–9.73)		

For DCC and intact UCM, there were 4 patients who died before discharge in each group, and for cut UCM there were three patients who died before discharge. In DCC group, the time to death was significantly shorter, however, the three groups did not differ significantly in term of pre-discharge mortality (S-Tables 2 and 3). Days to discharge were not significantly different between the studied groups (S-Table 1). S-Tables 4, 5, 6, 7, 8 and 9 show comparisons between echocardiographic parameters on day 1 and day 3 in each of the three groups.

## Discussion

A comparison of hemodynamics and cardiac functions in preterm infants with different placental transfusion techniques, I-UCM, C-UCM, and DCC, is presented in this study.

While placental transfusions have many benefits, the main disadvantage is the rapid transfusions provided by the 3 techniques and the possible changes in hemodynamics in vulnerable premature infants. This may not allow enough time for smooth transition and adaptation of cardiovascular system, especially in cord milking groups.

Another challenge that could be faced with cut cord milking is that blood would be transfused after taking the first breath and the available length of the cord segment to be milked might not be sufficient. However, this technique might be a good alternative if the two other techniques could not be done or missed.

No significant differences were found among studied groups in terms of demographics. In the DCC group, the type of delivery or anesthesia might affect the contractions of the uterus, and thus the amount of blood transfused. None of the studied groups demonstrated significant differences for those variables.

Katheria et al. found no differences in pulse oxygen saturation and heart rate between the UCM and DCC groups of infants delivered by cesarean section; their blood pressure was higher in UCM group in the first 15 h of life [13]. According to the present study, no significant differences were detected between the three studied groups in mean blood pressure, heart rate, or saturation in the first 24 h, nor were there any significant differences in temperature at admission. This might be attributed to short duration of procedure in intact cord milking, transfusion of warm blood in the DCC, and the use of servo-controlled heater during cut cord milking.

Whether different techniques of placental transfusion have similar effect on hemoglobin (Hb) level is point of debate in various studies. Kilicdag and Shirk showed no significant differences regarding Hb level between C-UCM and I-UCM, and DCC and I-UCM, respectively [18, 19]. Katheria et al. showed significant difference in Hb levels between DCC and I-UCM in preterm infants born by C-section, in which infants in DCC group are expected to have lower Hb levels owing to lack of effective uterine contractions in their mothers [13]. In the current work, the mean values of hemoglobin in the three groups were more than 15 g/dl. Although the results were not statistically significant, the I-UCM group had a tendency toward higher hemoglobin levels and fewer newborns with hemoglobin concentrations below 15 mg/dl, which also suggests that I-UCM may be a better choice in premature neonates.

An observation was made in the DCC group that 29 weeker male patient had the lowest initial hemoglobin level (10 mg/dl) and the highest WBCs (80 × 103 µl), the umbilical cord was noted as being pale and whitish in color and had a very little amount of blood to be transfused at birth. However, the patient was not excluded because he had none of the exclusion criteria. Right IVH grade I was noted on day 1 with SVC was 171.7 ml/kg/min, LVO was 397 ml/kg/min, RVO 409 ml/kg/min ductal shunt was unrestricted on the first day of life, and the day 3-SVC flow decreased to 90.3 ml/kg/min on the third day after receiving packed cell transfusion on day 2.

The lowest SVCF is usually recorded around 3–12 h after birth, with a gradual increase thereafter until 24–48 h after birth. After the transitional phase, the SVC flow remains relatively stable at 85–90 ml/kg/min in preterm infants [20]. In the first 24 h, placental transfusion might be one of principal factors affecting hemodynamics and myocardial function in the study population. Metrics of the 2nd scan, on day 3 might represent partial recovery from effect of placental transfusion and might be a reflection of other postnatal factors such as sepsis-related illness, mechanical ventilation, and the use of inotropes [21].

SVCF was significantly higher in the DCC group in the first 24 h after birth than I-UCM and C-UCM groups. This difference disappeared on day 3. The relative increase in SVCF in the 1st 24 h might played a role in lack of occurrence of catastrophic IVH in the 3 studied group in first day of life. Moreover, only 2 patients developed IVH grade I in the DCC group with significantly higher mean values of SVCF. SVCF is a measure of systemic blood flow (SBF) as well as RVO and LVO. Therefore, patients in the DCC group tended to have higher RVO and LVO.

In the current study, we used TAPSE, and EF and FS to evaluate systolic function of right and left ventricles, respectively. Left ventricular diastolic function was evaluated using E/A ratio.

There is scarcity of data about normative and reference values of echocardiographic parameters in preterm infants [22]. Noori et al. in their observational study on 29 preterm infants with gestational age = 26.2 ± 1.5 weeks reported that average FS was 34% ± 5% (range 23–48%) in the first 3 postnatal days in hemodynamically stable preterm infants [23]. Left ventricular dysfunction can be classified into the following: mild (FS 20–25%), moderate (FS 15–19%), and severe (FS ≤ 14%) [22]. In the current work, 8/19, 8/19, and 10/19 patients had FS less than 23% in C-UCM, I-UCM, and DCC, respectively. However, 2 out of those 26 patients only needed inotropic support and only three of them have LVO < 120 ml/kg /min; the normal LVO range in neonates is 150–400 ml/kg/min [24]. This might indicate that normative values might need be adjusted to a lower level in premature infants. It is also probably that rapid autotransfusion by the three placental transfusion techniques might have affected left ventricle (LV) systolic function. Taking into consideration that FS and EF measured by linear left ventricular minor-axis dimensions reflect movement of region of LV along the cursor line of M-mode, so it underestimates the entire LV contractile function.

The LV E/A ratio values were normal in the three study groups since the lower accepted value is 0.7:1 in premature infants [25] and no significant differences were found between the three groups. TAPSE was significantly lower in DCC group despite still being within physiological range of premature infants and this difference disappeared on day 3. The lowest accepted value for TAPSE in premature neonates is 4 mm [26]. This finding is similar to what happens in recipient fetus in twin to twin (TTT) syndrome with more affection of right ventricle by volume overload. After fetoscopic laser coagulation of the connecting vessel, rapid normalization of cardiac function occurs [27].

Early and rapid volume expansion might have detrimental effect on ventricular functions in preterm infants [28]. The immature myocardium has limited ability to tolerate over transfusion or rapid transfusions with decreased contractile reserve, as only 30% of the fetal myocardium contains contractile mass, in contrast to 60% in the adult [29]. The right ventricle (RV) is more vulnerable to transfusion intolerance. RV has a higher resting tension than the adult RV, leading to lower ventricular compliance in utero and early neonatal life [29]. The coronary flow is related to pressure difference between aortic root to the right atrium (RA). When RA pressure escalates, thus, the flow in right coronary artery might be insufficient leading to transient wall ischemia [30]. In the current work, DCC patients’ right ventricles were affected more than the other 2 groups. This could have 2 possible explanations: first, the amount of transfused blood might be higher in DCC patients. DCC for 30–45 s in preterm infants resulted in up to 28 ml/kg increase in blood volume, while C-UCM with 30-cm cord segment and I-UCM for 4 times may increase blood volume by 8 ml/kg and 14 ml/kg, respectively [31]. Second, patients of DCC group have lower birthweight and gestational age (Table 1) and TAPSE values increase linearly with gestational age and birth weight [32]. Yao et al. suggested that late cord clamping, by allowing a sizable placental transfusion, appeared to affect adversely the LV performance of the neonates [33].

Among neonatologists, there is still no consensus regarding when the umbilical cord should be clamped in preterm infants. Two systematic reviews of 11,736 preterm infants, encompassing more than 100 studies, found that delayed cord clamping was associated with lower mortality than immediate cord clamping [34, 35]. On the other hand, a large multicenter RCT for 1566 preterm infants found that delayed cord clamping did not result in a lower incidence of death or major morbidity at 36 weeks of gestation when compared to immediate cord clamping [36]. While in their long-term follow-up analysis of this mega multicenter RCT revealed that clamping the umbilical cord ≥ 60 s after birth resulted in a 30% reduction in relative mortality without affecting the major disability [37]. In the current study, there were no differences regarding predischarge mortality among the study groups, but the DCC group had lower days to death than the other 2 groups. The small sample size was a major limitation that hampers taking this result into consideration, especially that the DCC group had lower GA and birth weight than the other two groups.

## Limitations

In our trial, we were limited by the lack of an immediate cord clamping group. This might justify absence of significant differences in most of echocardiographic parameters between the three evaluated groups, because these three approaches resulted in increased SBFs and altered hemodynamics. It was also important to note that the sample size of the three groups was relatively small in the present study.

The study was also limited by the inability to measure the SVC flow and cardiac output more than two times, since the SVC flow tends to vary widely during the first days of life. A further limitation of the study was the fact that no actions were taken when the SVC flow decreased, as the study was designed as a prospective cohort study, regarding the imaging step. We did not evaluate diastolic function of RV that might be affected by placental transfusions more than diastolic function left ventricle.

## Conclusion

Although there are benefits of different placental transfusion techniques, they appear to alter hemodynamics and myocardial function in premature neonates ≤ 32 weeks. Use of delayed cord clamping technique results in significantly increased SVCF and decreased right ventricular systolic function in premature neonates in their 1st 24 h of life. This effect disappeared on day 3. Therefore, although DCC is preferred method because its preferable effect on SVCF in the 1st 24 h, patient’s myocardial function should be monitored. It is possible that the myocardium, especially in patients with delayed cord clamp, may be more susceptible to potential adverse cardiovascular effect than premature neonates without placental transfusion.

## Supplementary Information

Below is the link to the electronic supplementary material.

Supplementary file1 (DOCX 44 KB)

## Data Availability

The datasets generated during and/or analyzed during the current study are available from the corresponding author on reasonable request.
