# Supplementary material for: The effect of placental transfusion on hemodynamics in premature newborns: a randomized controlled trial
Source: Eur J Pediatr. 2022 Sep 21;181(12):4121–33. doi: 10.1007/s00431-022-04619-0 (PMC9649456; doi:10.1007/s00431-022-04619-0)
Supplement: Supplementary file 1 — Supplementary file1 (DOCX 44 KB) [file 431_2022_4619_MOESM1_ESM.docx]

**Online supplements**

**S-table1: Duration of hospital stays among survived patients in the three studied groups.**

|  | Cut Umbilical Cord  Milking  (No.=16) | DCC  (No.=15) | Intact UCM  (No.=15) | **Test of significance**  **(P)** |
| --- | --- | --- | --- | --- |
| Time to discharge from hospital (Days) | 36.5(24-90) | 43(15-66) | 39(14-75) | (H=.327,P=.849) |

H;Kruskal-Wallis test

**S-table2: Days to death in the three studied groups.**

|  | Cut Umbilical Cord  Milking  (No.=3) | DCC  (No.=4) | Intact UCM  (No.=4) | **Test of significance**  **(P)** |
| --- | --- | --- | --- | --- |
| Time to Death (Days) | 33(9-38) | 5.5(5-11) | 24(14-31) | (H=6.2,P=.045*) |
| Sig .between groups | Sig between (Cut Umbilical Cord Milking Vs DCC) & (DCC Vs Intact UCM) .( P=(.027&.042 respectively) | | |  |

H;Kruskal-Wallis test for significant results, pair wise comparison was done

| **Test of significance** | **Cut UCM**  **(No.=19)** | | **DCC**  **(No.=19)** | | **Intact UCM**  **(No.=19)** | |  |
| --- | --- | --- | --- | --- | --- | --- | --- |
| **MCP=1** | **%** | **No.** | **%** | **No.** | **%** | **No.** |  |
|  | **21.1** | **4** | **21.1** | **4** | **15.8** | **3** | **Death** |
|  | **78.9** | **15** | **78.9** | **15** | **84.2** | **16** | **Discharged to home** |

**S-Table 3: Comparison between the three groups regarding predischarge deaths**

**S-Table 4: Comparison between echocardiographic parameters of Cut UCM group in the 1^st^ and second scans.**

| **ECHO**  **Parameters**  **Cut CM group** | **1^st^ time** | **2^nd^ time** | **Test of significance**  **(p)** |
| --- | --- | --- | --- |
| **SVC Flow (ml/kg/min):** |  |  | z= -1.24  p= 0.212 |
| Mean ± SD | 83.5 ± 34.83 | 92.92± 24.99 |  |
| Median (Min – Max) | 72.4 (36.3 – 173.7) | 98.7(44.4– 138) |  |
| **LA/AO ratio:** |  |  | z= -1.7  p= 0.08 |
| Mean ± SD | 1.28 ± 0.32 | 1.14 ± 0.16 |  |
| Median (Min – Max) | 1.2 (0.8 – 2.0) | 1.1 (1 – 1.47) |  |
| **EF (%):** |  |  | z= -0.632  p= 0.52 |
| Mean ± SD | 48.84 ± 7.17 | 50.05 ± 10.2 |  |
| Median (Min – Max) | 50.0 (37.0 – 60.0) | 51 (30 – 70.0) |  |
| **FS (%):** |  |  | z= -0.69  p= 0.49 |
| Mean ± SD | 22.37 ± 3.92 | 23.15± 5.7 |  |
| Median (Min – Max) | 23.0 (16.0 – 29.0) | 23.0 (13 – 36) |  |
| **LVO:** |  |  | z= -0.724  p= 0.46 |
| Mean ± SD | 192.24 ± 58.18 | 181.9 ± 48.3 |  |
| Median (Min – Max) | 177.7 (110.5 – 311) | 171 (112.6 – 313.3) |  |
| **RVO:** |  |  | z= -1.6  p= 0.09 |
| Mean ± SD | 270.32 ± 69.52 | 309.2± 79.4 |  |
| Median (Min – Max) | 263 (155.8 – 429) | 306.6 (191.4– 468) |  |
| **E/A Ratio:** |  |  | z= -0.93  p= 0.355 |
| Mean ± SD | 1.04 ± 0.21 | 1 ± 0.11 |  |
| Median (Min – Max) | 1.01 (0.8 – 1.74) | 1 (0.8 – 1.26) |  |
| **TAPSE (**[**101**](#_ENREF_101)**):** |  |  | z= -1.6  p= 0.11 |
| Mean ± SD | 0.53 ± 0.07 | 0.5 ± 0.06 |  |
| Median (Min – Max) | 0.5 (0.4 – 0.6) | 0.5 (0.4 – 0.6) |  |

**z; Wilcoxon Ranked test**

**S-Table 5: Comparison between Comparison between PDA parameters of Cut UCM group in the 1^st^ and second scans.**

| **ECHO parameters** | **1^st^ time** | | | | **2^nd^ time** | | **Test of significance**  **(p)** |
| --- | --- | --- | --- | --- | --- | --- | --- |
|  | **No.** | | **%** | | **No.** | **%** |  |
| **PDA closure:** |  | |  | |  |  | ^MC^p= 0.0015* |
| Closed | 4 | | 21.1 | | 15 | 78.9 |  |
| Non hs-PDA | 9 | | 47.3 | | 3 | 15.8 |  |
| Hs-PDA | 6 | | 31.6 | | 1 | 5.3 |  |
| **Size of PDA (mm):** | |  | |  | | | z= -1.46  p= 0.144 |
| Mean ± SD | | 1.31 ± 0.95 | | 1.2 ± 0.46 | | |  |
| Median (Min – Max) | | 1.36 (0.0 – 3.64) | | 1.25 (0.6 – 1.7) | | |  |
| **Shunt Direction:** |  | |  | |  |  | ^MC^p= 0.0006* |
| None | 4 | | 21.1 | | 15 | 78.9 |  |
| Bidirectional | 0 | | 0.0 | | 0 | 0.0 |  |
| Left to right restrictive | 8 | | 42.1 | | 4 | 21.1 |  |
| Left to right non-restrictive | 7 | | 36.8 | | 0 | 0.0 |  |

**^MC^p; Monte Carlo Exact Probability**

**S-Table 6:** **Comparison between echocardiographic parameters of intact-UCM group in the 1^st^ and second scans.**

| **ECHO**  **Parameters**  **Intact CM group** | **1^st^ time** | **2^nd^ time** | **Test of significance**  **(p)** |
| --- | --- | --- | --- |
| **SVC Flow (ml/kg/min):** |  |  | z= -0.523  p= 0.601 |
| Mean ± SD | 97.44 ± 30.27 | 96.4± 40.9 |  |
| Median (Min – Max) | 91.7 (60 – 170) | 82.5(29 – 180.7) |  |
| **LA/AO ratio:** |  |  | **z= -2.069**  **p= 0.039*** |
| Mean ± SD | 1.18 ± 0.15 | 1.1 ± 0.21 |  |
| Median (Min – Max) | 1.2 (1.0 – 1.5) | 1.07 (.8– 1.8) |  |
| **EF (%):** |  |  | z= -0.392  p= 0.695 |
| Mean ± SD | 50.0 ± 7.51 | 50.05 ± 8.99 |  |
| Median (Min – Max) | 51.0 (34.0 – 64.0) | 49 (34.0 – 68) |  |
| **FS (%):** |  |  | z= -0.327  p= 0.743 |
| Mean ± SD | 22.95 ± 4.17 | 22.7 ± 5.18 |  |
| Median (Min – Max) | 23.0 (14.0 – 31.0) | 22 (14.0 – 34) |  |
| **LVO:** |  |  | z= -0.201  p= 0.841 |
| Mean ± SD | 162.16 ± 41.0 | 169.8 ± 72 |  |
| Median (Min – Max) | 159 (78.8 – 259.4) | 160 (71.7 – 360) |  |
| **RVO:** |  |  | z= -1.932  p= 0.053 |
| Mean ± SD | 327.36 ± 104.55 | 401.7 ± 162.47 |  |
| Median (Min – Max) | 336.6 (161 – 538.8) | 397.6(124.4 – 759) |  |
| **E/A Ratio:** |  |  | z= -0.310  p= 0.756 |
| Mean ± SD | 0.96 ± 0.12 | 0.98 ± 0.14 |  |
| Median (Min – Max) | 0.92 (0.7 – 1.27) | 1 (0.6 – 1.26) |  |
| **TAPSE (**[**101**](#_ENREF_101)**):** |  |  | z= -1.567  p= 0.117 |
| Mean ± SD | 0.47 ± 0.08 | 0.44 ± 0.08 |  |
| Median (Min – Max) | 0.5 (0.4 – 0.6) | 0.5 (0.2 – 0.5) |  |

**z; Wilcoxon Ranked test**

**S-Table 7: Comparison between PDA parameters of intact-UCM group in the 1^st^ and second scans.**

| **ECHO parameters** | | **1^st^ time** | | **2^nd^ time** | | **Test of significance**  **(p)** | |
| --- | --- | --- | --- | --- | --- | --- | --- |
|  |  | **No.** | **%** | **No.** | **%** |  |  |
| **PDA closure:** | |  |  |  |  | ^MC^p= 0.003* | |
| Closed | | 10 | 52.6 | 19 | 100 |  |  |
| Non hs-PDA | | 6 | 31.6 | 0 | 0 |  |  |
| Hs-PDA | | 3 | 15.8 | 0 | 0 |  |  |
| **Size of PDA (mm):** |  | | |  | | |  |
| Mean ± SD | 0.67 ± 1.01 | | |  | | |  |
| Median (Min – Max) | 0.0 (0.0 – 3.8) | | |  | | |  |
| **Shunt Direction:** | |  |  |  |  | ^MC^p= 0.008* | |
| None | | 10 | 52.6 | 19 | 100 |  |  |
| Bidirectional | | 1 | 5.3 | 0 | 0 |  |  |
| Left to right restrictive | | 6 | 31.6 | 0 | 0 |  |  |
| Left to right non-restrictive | | 2 | 10.5 | 0 | 0 |  |  |

**^MC^p; Monte Carlo Exact Probability**

**S-Table 8: Comparison between echocardiographic parameters of DCC group in the 1^st^ and second scans.**

| **ECHO**  **Parameters**  **Intact CM group** | **1^st^ time** | **2^nd^ time** | **Test of significance**  **(p)** |
| --- | --- | --- | --- |
| **SVC Flow (ml/kg/min):** |  |  | z= -0.805  p= 0.421 |
| Mean ± SD | 120.29 ± 43.11 | 103.99 ± 40.9 |  |
| Median (Min – Max) | 99.0 (55 – 183.6) | 96.5(46.3 – 220) |  |
| **LA/AO ratio:** |  |  | z= -0.624  p= 0.532 |
| Mean ± SD | 1.22 ± 0.24 | 1.23 ± 0.3 |  |
| Median (Min – Max) | 1.14 (0.97 – 1.7) | 1.1 (0.86 – 2.2) |  |
| **EF (%):** |  |  | z= -1.613  p= 0.107 |
| Mean ± SD | 47.32 ± 7.82 | 51 ± 13.7 |  |
| Median (Min – Max) | 46.0 (29.0 – 61.0) | 50 (30 – 77) |  |
| **FS (%):** |  |  | z= -1.328  p= 0.184 |
| Mean ± SD | 21.53 ± 4.31 | 23.8 ± 8.4 |  |
| Median (Min – Max) | 21.0 (12.0 – 30.0) | 23 (12.0 – 42) |  |
| **LVO:** |  |  | z= -0.845  p= 0.398 |
| Mean ± SD | 216.15 ± 89.8 | 194.59 ± 87.9 |  |
| Median (Min – Max) | 203.8  (83.8 – 397) | 183.3  (90 – 478.5) |  |
| **RVO:** |  |  | z= -0.282  p= 0.778 |
| Mean ± SD | 331.64 ± 89.4 | 345.3 ± 97.84 |  |
| Median (Min – Max) | 335.8  (191.4 – 463.3) | 354  (196 – 583.7) |  |
| **E/A Ratio:** |  |  | z= -0.283  p= 0.777 |
| Mean ± SD | 0.92 ± 0.16 | 0.96 ± 0.25 |  |
| Median (Min – Max) | 0.9 (0.57 – 1.17) | 0.9 (0.6 – 1.7) |  |
| **TAPSE (**[**101**](#_ENREF_101)**):** |  |  | z= -1.5  p= 0.134 |
| Mean ± SD | 0.45 ± 0.06 | 0.48 ± 0.06 |  |
| Median (Min – Max) | 0.4 (0.4 – 0.6) | 0.5 (0.4 – 0.6) |  |

z; Wilcoxon Ranked test

**S-Table 9: Comparison between PDA parameters of DCC group in the 1^st^ and second scans.**

| **ECHO parameters** | | **1^st^ time** | | | **2^nd^ time** | | **Test of significance**  **(p)** |
| --- | --- | --- | --- | --- | --- | --- | --- |
|  |  | **No.** | **%** | | **No.** | **%** |  |
| **PDA closure:** | |  |  | |  |  | ^MC^p= 0.055 |
| Closed | | 8 | 42.1 | | 15 | 78.9 |  |
| Non hs-PDA | | 5 | 26.3 | | 1 | 5.3 |  |
| Hs-PDA | | 6 | 31.6 | | 3 | 15.8 |  |
| **Size of PDA (mm):** |  | | |  | | | z= -1.095  p= 0.273 |
| Mean ± SD | 1.21 ± 1.1 | | | 1.8 ± 0.78 | | |  |
| Median (Min – Max) | 1.3 (0.0 – 3.0) | | | 1.8 (1 – 2.9) | | |  |
| **Shunt Direction:** | |  |  | |  |  | ^MC^p= 0.006* |
| None | | 7 | 36.8 | | 15 | 78.9 |  |
| Bidirectional | | 0 | 0.0 | | 2 | 10.5 |  |
| Left to right restrictive | | 4 | 21.1 | | 0 | 0.0 |  |
| Left to right non-restrictive | | 8 | 42.1 | | 2 | 10.5 |  |

^MC^p; Monte Carlo Exact Probability
